# Metagenomic analysis reveals rumen microbiome enrichment and functional genes adjustment in carbohydrate metabolism induced by different sorting behavior in mid-lactation dairy cows

**DOI:** 10.1186/s42523-025-00439-3

**Published:** 2025-07-28

**Authors:** Abdallah Alaa Mousa, Han Zhang, Hongwei Duan, Jiyou Zhang, Shengyong Mao

**Affiliations:** 1 Ruminant Nutrition and Feed Engineering Technology Research Center, College of Animal Science and Technology, Nanjing Agricultural University, Nanjing, 210095 Jiangsu China; 2 Laboratory of Gastrointestinal Microbiology, College of Animal Science and Technology, Nanjing Agricultural University, Nanjing, 210095 China; 3 Animal Production Department, Faculty of Agriculture, Benha University, Benha, 13736 Egypt

**Keywords:** Feed sorting, Microbiome, Rumen fermentation, Digestibility, KEGG modules, Dairy cow

## Abstract

**Background:**

This study aimed to investigate differences in the structure and function of the rumen microbiome and its associated changes in rumen fermentation patterns and apparent nutrient digestibility in dairy cattle with different sorting behavior. Twenty-four Holstein cows in mid-lactation were initially enrolled in the experiment. All cows were fed and milked three times daily throughout the entire 28-day experimental period, comprising a 7-day pre-trial and a 21-day main trial. On days 1, 7, 14, and 21 of the main trial, feed sorting behavior was measured, and feed and feces samples were collected to determine apparent nutrient digestibility. Rumen content samples were collected on day 21 to measure pH, volatile fatty acids (VFA), and rumen microbiome structure and function. Based on feed sorting behavior, twelve cows were selected and divided into two groups: six cows that were severely sorted for fine particles-severely rejected long particles (SES; *n* = 6) and six cows that were slightly sorted for fine particles-slightly rejected long particles (SLS; *n* = 6).

**Results:**

Comparative analysis revealed significant differences between the groups. The SES group exhibited lower rumen pH values and higher concentrations of total VFA (TVFA) and acetate (*P* < 0.05) than the SLS group. Data on apparent nutrient digestibility showed that compared to the SLS group, the SES group lowered the digestibility of neutral detergent fiber (NDF) and acid detergent fiber (ADF) (*P* < 0.05). Differential analysis of rumen microbiota indicated that the SES group had a higher relative abundance of *Prevotella*, *Lactobacillus*, *Bifidobacterium*, *Selenomonas*, and *Acetitomaculum* by a lower relative abundance of *Fibrobacter*, *Ruminobacter*, *Pseudobutyrivibrio*, *Butyrivibrio*, and *Ruminococcus*. Carbohydrate-active enzyme (CAZyme) annotation revealed that the SES group showed increased abundance of GH13 and GH65 enzymes, while exhibiting decreased abundance of GH1, GH3, GH5, GH6, and GH94. Functional profiling of Kyoto encyclopedia of genes and genomes (KEGG) modules revealed that compared to the SLS group, the rumen microbiota in the SES group upregulated the abundance of carbohydrate metabolism, amino acid metabolism, energy metabolism, and lipid metabolism. In carbohydrate metabolism, the rumen microbiota in the SES group upregulated the abundance of starch and sucrose metabolism, the citrate cycle, and pyruvate metabolism, while downregulating the pentose phosphate pathway. Functional profiling of KEGG Orthology (KO) enzymes revealed that the microbiota in the SES group preferred energy production through increasing glycolysis and supported the metabolism changes toward acetate production and fatty acid biosynthesis.

**Conclusion:**

Our findings reveal that feed sorting behavior significantly alters the rumen microbial ecosystem and its metabolic functions, negatively impacting fermentation efficiency, fiber digestibility, and overall nutrient utilization, even when cows are provided a well-balanced, standardized diet. This underscores the importance of early detection and management of feed sorting in dairy farms to promote cows’ health and support sustainable dairy production.

**Graphical abstract:**

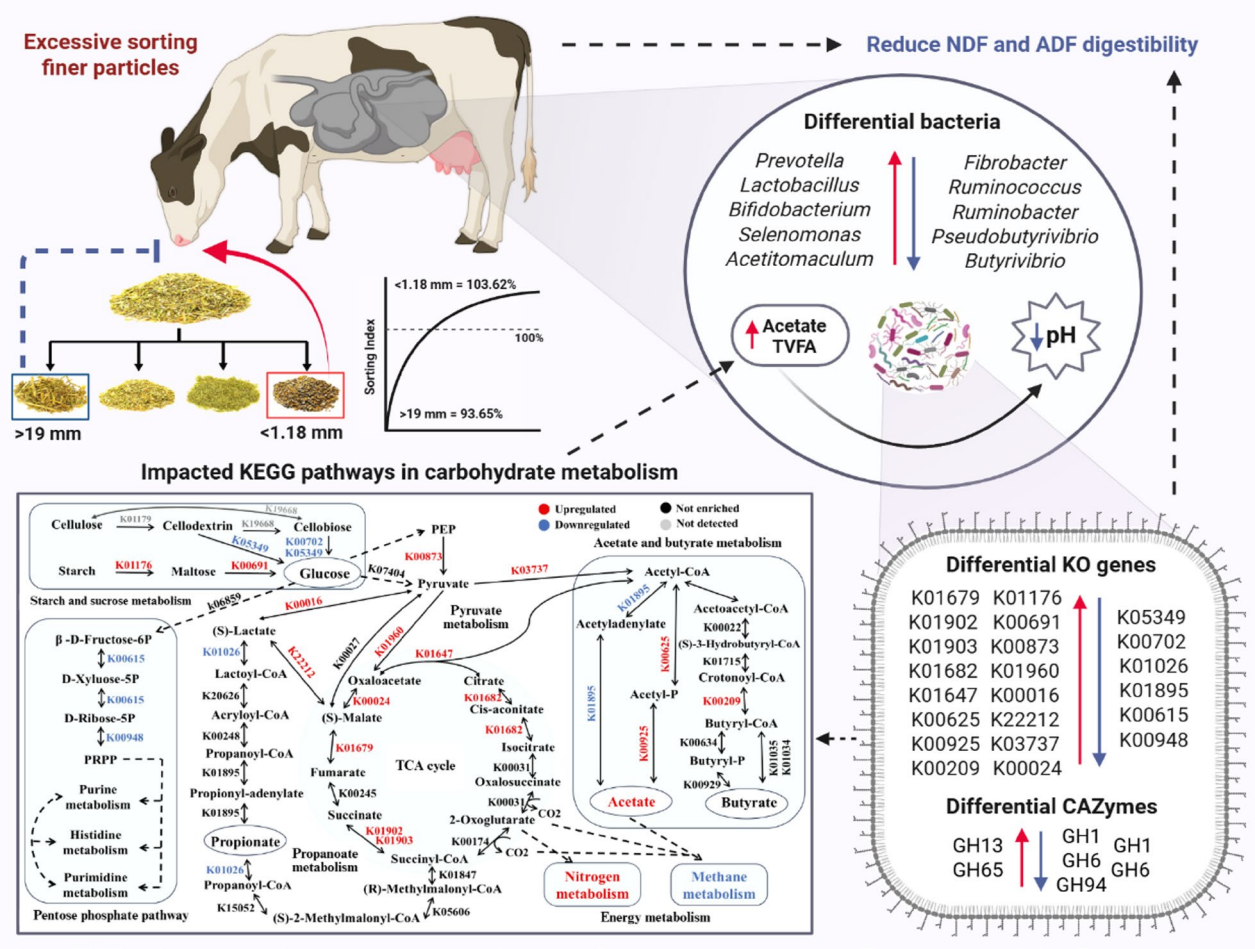

**Supplementary Information:**

The online version contains supplementary material available at 10.1186/s42523-025-00439-3.

## Background

Formulating a balanced total mixed ration (TMR) is fundamental for ensuring the health, productivity, and metabolic stability of dairy cows. TMR is designed to provide a uniform blend of forages and concentrates, precisely tailored to meet the nutritional requirements of dairy cows while promoting optimal rumen function and stabilizing rumen pH [1]. However, the effectiveness of this feeding strategy is often compromised by feed sorting behavior. Dairy cows have an intrinsic ability to separate grains from forage in TMR, leading them to selectively consume grains while rejecting forage components [2]. As a result, they ingest more non-structural carbohydrates and less physically effective neutral detergent fiber (peNDF) than intended in the TMR formula [3]. This behavior can be particularly detrimental when cows excessively sort TMR for finer particles, as it disrupts the uniformity of nutrient intake, alters substrate availability in the rumen, and destabilizes rumen fermentation patterns. Such disturbances can result in subacute ruminal acidosis (SARA) and other metabolic disorders [4], ultimately impairing cow health, milk production, and overall herd productivity.

The diet–microbiome–host interaction forms a complex symbiotic relationship that has attracted considerable interest in recent decades due to its profound implications for health and disease [5, 6]. Rumen microbiota, which are intimately related to dietary composition and host metabolism, can be impacted by diet-related factors such as pH and substrate availabilities in the rumen [7]. As mentioned earlier, feed sorting, especially excessive sorting for fine particles in TMR, can significantly alter the substrate availability in the rumen, potentially disrupting the rumen microbiota composition and function, affecting key microbial enzymatic activities, and associated metabolic pathways in the rumen. To date, limited studies on such interaction in grain-based diets have been conducted in dairy cows [8]. However, how feeding behavior, especially excessive sorting for fine particles within a balanced TMR, can alter rumen microbiome structure and function remains largely unexplored.

The digestibility of nutrients in dairy cows is heavily influenced by the rumen microbiota, which plays a critical role in digesting starch and fiber into absorbable nutrients [9]. Microbial populations, including amylolytic and fibrolytic bacteria, adapt to dietary changes, with grain-based diets enriching starch-digesting microbiota while reducing fiber-degrading microbiota [10]. Excessive sorting for fine particles potentially disrupts the microbiota’s structure and enzymatic functions, impairing the digestibility of NDF and ADF. Such changes highlight the potential role of rumen microbiota in modulating fiber digestibility in response to different sorting behavior, ultimately impacting nutrient utilization and rumen health.

We hypothesize that excessive sorting for fine particles disrupts the structure and function of the rumen microbiome, alters rumen fermentation patterns, and induces changes in apparent nutrient digestibility. Therefore, the objectives of this study were to investigate the implications of excessive sorting for fine particles in TMR on the rumen microbiome’s structure and function, as well as the associated changes in rumen fermentation patterns and apparent nutrient digestibility in dairy cows.

## Results

### Feed sorting index

Feed sorting indices indicated that both SES and SLS groups sorted TMR for fine particles (*P* < 0.001; Table 1), but the SES group sorted to a greater extent than the SLS group. Additionally, both groups refused long (*P* < 0.001), medium (*P* < 0.001), and short (*P* < 0.001) particles, but the SES group sorted against these particles to a greater extent (Table 1). The results showed that sorting time had no significant effect on the sorting behavior of each group, implying that all cows maintained consistent feed sorting behavior over the course of the experimental period.

**Table 1:** Feed sorting index of long, medium, short, and fine particles selected by the SES and SLS groups

Sorting index^1^	Groups		SEM	*P*-value		
	SES	SLS		Group	Day	Group*day
> 19 mm	93.65^b^	98.66^a^	1.101	< 0.001	0.156	0.403
8–19 mm	95.89^b^	99.38^a^	0.533	< 0.001	0.069	0.824
1.18–8 mm	97.53^b^	99.71^a^	0.371	< 0.001	0.822	0.822
< 1.18 mm	103.62^a^	100.52^b^	0.346	< 0.001	0.071	0.097

^1^Sorting index were calculated by taking the ratio of actual intake to expected intake for the particles that were retained on each layer of the Penn State Particle Separator. A sorting index above 100 signifies sorting in favor of specific particles, a sorting index below 100 indicates sorting against specific particles [70]. Mean values with different superscripts (a, b) differ significantly at *P* < 0.05

### Rumen pH, rumen fermentation, and nutrients digestibility

Data on ruminal pH showed that the SES group recorded lower rumen pH values than the SLS group (pH = 6.14 vs. 6.46; *P* = 0.013; Table 2). Data on VFA showed that compared with the SLS group, the SES group recorded higher concentrations of TVFA (*P* = 0.026) and acetate (*P* = 0.025) in the rumen, and no significant differences were found in the rest of the VFA portions between the groups. Additionally, no differences were observed in the percentage of all VFA fractions, nor in the acetate/propionate (A/P) ratio between the two groups (Table 2). Data on apparent nutrient digestibility showed that compared to the SLS group, the SES group lowered the digestibility of NDF (*P* = 0.017; Table 3) and ADF (*P* = 0.001; Table 3), and there were no significant differences observed between the groups in terms of dry matter (DM), organic matter (OM), crude protein (CP), and ether extract (EE) digestibility.

**Table 2:** Effect of excessive sorting for fine particles on rumen pH and rumen VFA concentrations

Item	Groups		SEM	*P*-value
	SES	SLS		
Ruminal pH	6.14^b^	6.46^a^	0.097	0.013
Acetate, mmol/L	79.49^a^	69.15^b^	3.925	0.025
Propionate, mmol/L	25.13	21.96	2.625	0.256
Butyrate, mmol/L	11.86	11.20	0.802	0.435
Isobutyrate, mmol/L	0.77	0.72	0.271	0.863
Valerate, mmol/L	1.10	0.99	0.094	0.259
Isovalerate, mmol/L	1.00	0.93	0.150	0.668
TVFA, mmol/L	119.37^a^	104.96^b^	5.513	0.026
Acetate, %	66.71	65.78	1.636	0.582
Propionate, %	20.89	21.02	1.943	0.950
Butyrate, %	9.98	10.64	0.576	0.286
Isobutyrate, %	0.64	0.69	0.238	0.844
Valerate, %	0.92	0.95	0.091	0.777
Isovalerate, %	0.83	0.90	0.163	0.674
A/P	3.25	3.23	0.369	0.958

TVFA = total volatile fatty acids, A/*P* = acetate/propionate ratio. Mean values with different superscripts (a, b) differ significantly at *P* < 0.05

**Table 3:** Effect of excessive sorting for fine particles on apparent nutrient digestibility (%)

Item	Groups		SEM	*P*-value
	SES	SLS		
DM	63.00	63.47	1.835	0.806
OM	68.67	70.62	1.082	0.106
CP	76.30	77.80	2.240	0.519
EE	65.85	63.15	5.345	0.626
NDF	53.01^b^	63.03^a^	3.507	0.017
ADF	48.64^b^	62.80^a^	2.934	0.001

DM = dry matter, OM = organic matter, CP = crude protein, EE = ether extract, NDF = neutral detergent fiber, ADF = acid detergent fiber. Mean values with different superscripts (a, b) differ significantly at *P* < 0.05

### Taxonomic classifications of ruminal microbiota

**Fig. 1:**
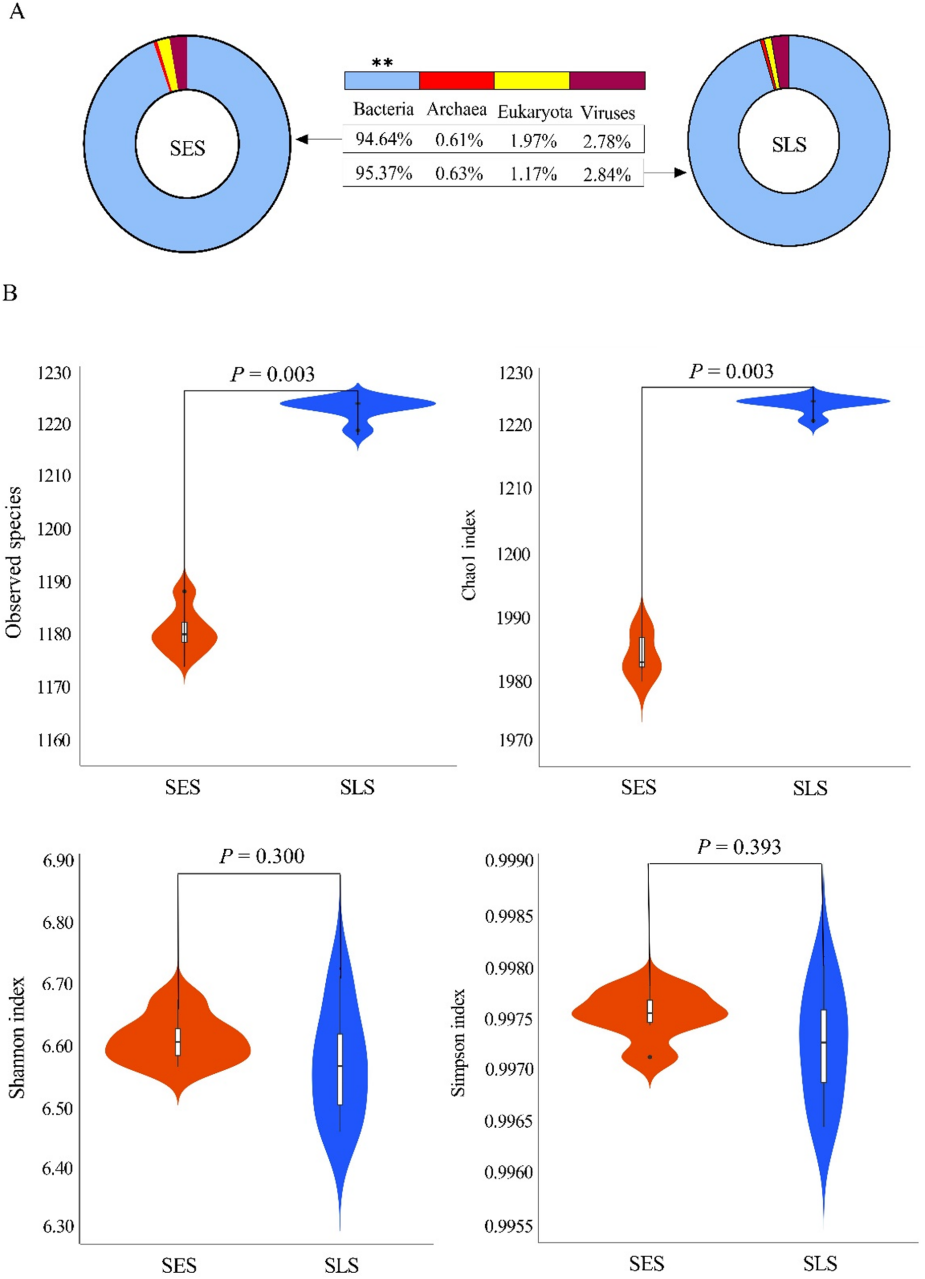
Differences in ruminal microbiota abundance and diversity between SES and SLS groups (**A**) Differences in the relative abundance of ruminal microbiota at the domain level; **P < 0.01. (**B**) Variations in alpha diversity indices (Observed species, Chao 1, Shannon, and Simpson) of ruminal bacterial communities between SES and SLS groups. The SES group is represented in red, while the SLS group is shown in blue (*n* = 6)

**Fig. 2:**
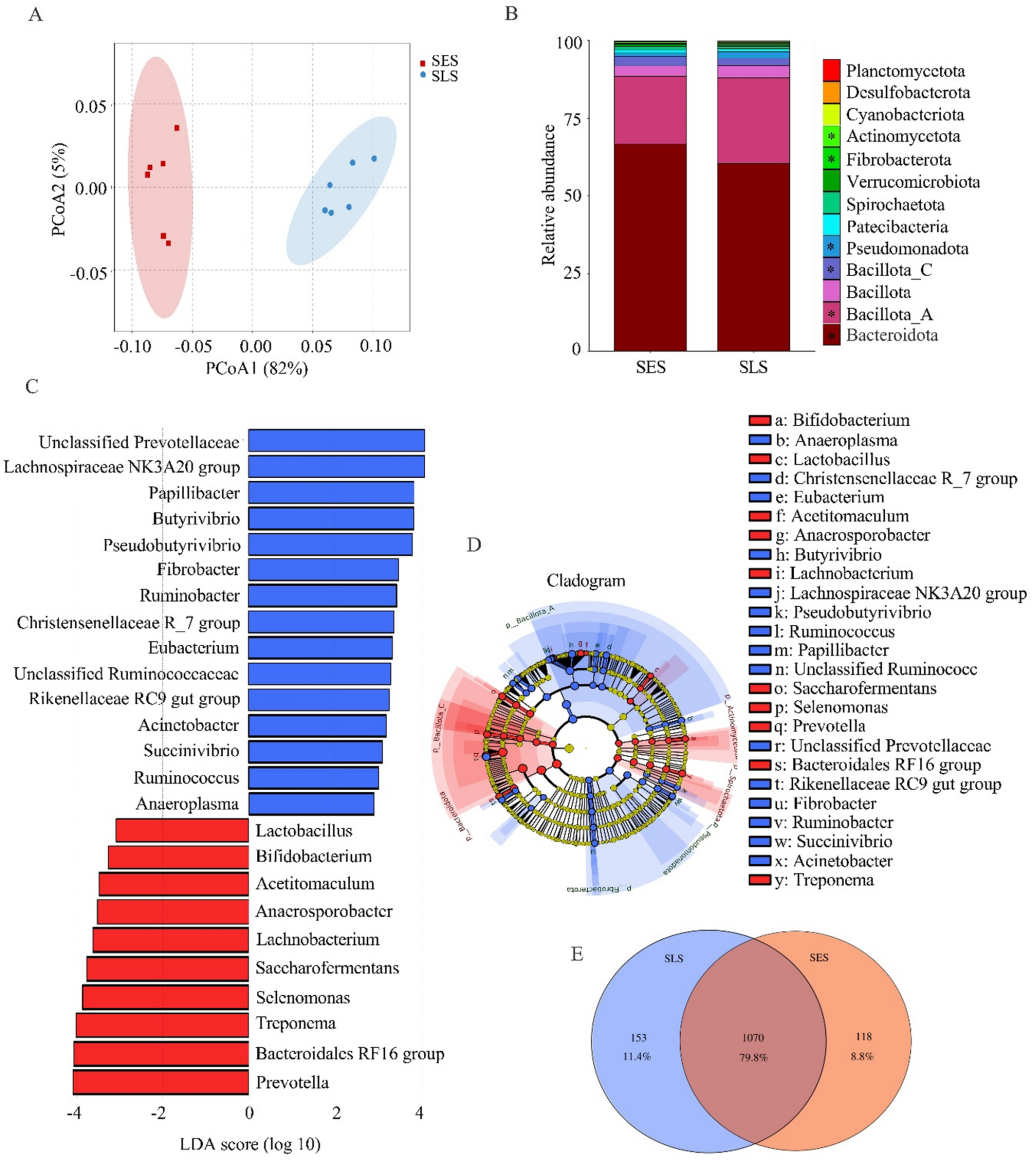
Changes of ruminal bacterial composition profiles between SES and SLS groups. (**A**) Principal Coordinate Analysis (PCoA) based on bacterial operational taxonomic units (OTUs), where the x-axis and y-axis represent the variance explained by PCoA1 and PCoA2, respectively. Shaded ellipses indicate 95% confidence intervals for each group. (**B**) Relative abundance of rumen bacteria at the phylum level; *P < 0.05. (**C**) Linear Discriminant Analysis (LDA) scores highlighting differentially abundant bacterial genera between SES and SLS groups (*P* < 0.05; LDA > 3). (**D**) Cladogram illustrating genus-level differences in bacterial abundance between the SES and SLS groups. (**E**) Venn diagram displaying the number of shared and unique species between the SES and SLS groups. The SES group is represented in red, while the SLS group is in blue (*n* = 6)

To investigate variations in microbial composition, four domains, including bacteria, archaea, eukaryota, and viruses, were detected in metagenomic samples. Statistical analysis revealed that the relative abundance of bacteria was significantly lower in the SES group compared to the SLS group (SES: 94.64% vs. SLS: 95.37%; *P* = 0.002). However, no statistically significant difference was observed in the relative abundance of archaea (SES: 0.61% vs. SLS: 0.63%), eukaryota (SES: 1.97% vs. SLS: 1.17%), or viruses (SES: 2.78% vs. SLS: 2.84%) between the groups (Fig. 1A). The analysis of ruminal bacterial communities revealed an average of 34,329 ± 3,181 reads per sample through gene sequencing. Further analysis of ruminal bacterial communities using a principal coordinates analysis (PCoA) plot based on the Bray-Curtis metric demonstrated clear segregation and dissimilarities between the two groups (Fig. 2A), indicating distinct bacterial community structures. Alpha diversity analysis indicated that the SES group exhibited significantly lower bacterial richness compared to the SLS group, as evidenced by the observed species (*P* = 0.003) and Chao1 indices (*P* = 0.003). However, no significant differences were observed in bacterial evenness, as indicated by the Shannon index (*P* = 0.300) and the Simpson index (*P* = 0.393) (Fig. 1B).

At the phylum level, we only listed the top 13 bacterial phyla, whose relative abundance was more than 0.1% in at least one group (Fig. 2B). Most sequences were allocated into *Bacteroidota* (SES = 66.77%; SLS = 59.85%), *Bacillota_A* (SES = 21.83%; SLS = 27.35%), *Bacillota_C* (SES = 3%; SLS = 2.25%), and *Bacillota* (SES = 3.84%; SLS = 3.92%). The differential analysis of the relative abundance (*P* < 0.05) revealed that 6 phyla were affected by excessive sorting for fine particles. Among them, the relative abundance of *Bacteroidota*, *Bacillota_C*, and *Actinomycetota* increased, whereas the relative abundance of *Bacillota_A*, *Fibrobacterota*, and *Pseudomonadota* decreased in the SES group (*P* < 0.05; Fig. 2B).

At the genus level, we listed the 30 bacterial genera with relative abundance more than 0.1% in at least one group (Fig.S1). Among them, *Prevotella* was the most predominant genus in the rumen (SES = 35.70%; SLS = 32.62%). Differential analysis (*P* < 0.05; LDA > 3) revealed that 25 genera were affected between groups (Fig. 2C and D). The relative abundances of *Prevotella*, *Lactobacillus*, *Bifidobacterium*, *Selenomonas*, *Treponema*, *Lachnobacterium*, *Saccharofermentans*, *Bacteroidales RF16 group*, *Anaerosporobacter*, and *Acetitomaculum* were higher in the SES group. Moreover, the relative abundances of *Fibrobacter*, *Ruminobacter*, *Pseudobutyrivibrio*, *Butyrivibrio*, *Papillibacter*, *Lachnospiraceae NK3A20* group, *Unclassified Prevotellaceae*, *Christensenellaceae R_7 group*, *Eubacterium*, *Unclassified Ruminococcaceae*, *Rikenellaceae RC9 gut group*, *Acinetobacter*, *Succinivibrio*, *Ruminococcus*, and *Anaeroplasma* were lower in the SES group.

At the OTU (operational taxonomic unit) level, the Venn analysis indicated that the SES and SLS groups had 118 (8.8%) and 153 (11.4%) unique OTUs, respectively, and both groups shared 1070 (79.8%) bacterial species (Fig. 2E). We filtered 189 OTUs that exhibited a relative abundance of more than 0.1% in at least one group. The differential analysis (*P* < 0.05) revealed that 38 OTUs were changed between the SES and SLS groups. Among them, the relative abundance of *Prevotella albensis*,* Prevotella bryantii*,* Prevotella ruminicola*,* Prevotella brevis*,* Lactobacillus acidophilus*,* Treponema bryantii*,* Selenomonas ruminantium*,* Saccharofermentans acetigenes*,* Lachnobacterium bovis*,* Bifidobacterium adolescentis*,* Bifidobacterium merycicum*, and *Acetitomaculum ruminis* were increased in the SES group. Additionally, the relative abundances of *Butyrivibrio fibrisolvens*,* Butyrivibrio proteoclasticus*,* Fibrobacter succinogenes*,* Papillibacter cinnamivorans*,* Pseudobutyrivibrio xylanivorans*,* Ruminobacter amylophilus*,* Eubacterium cellulosolvens*,* Acinetobacter baumannii*,* Ruminococcus albus*,* Ruminococcus flavefaciens*, and *Anaeroplasma bactoclasticum* were decreased in the SES group compared to the SLS group (Table S1).

### Changes in KEGG modules in response to different sorting behavior

**Fig. 3:**
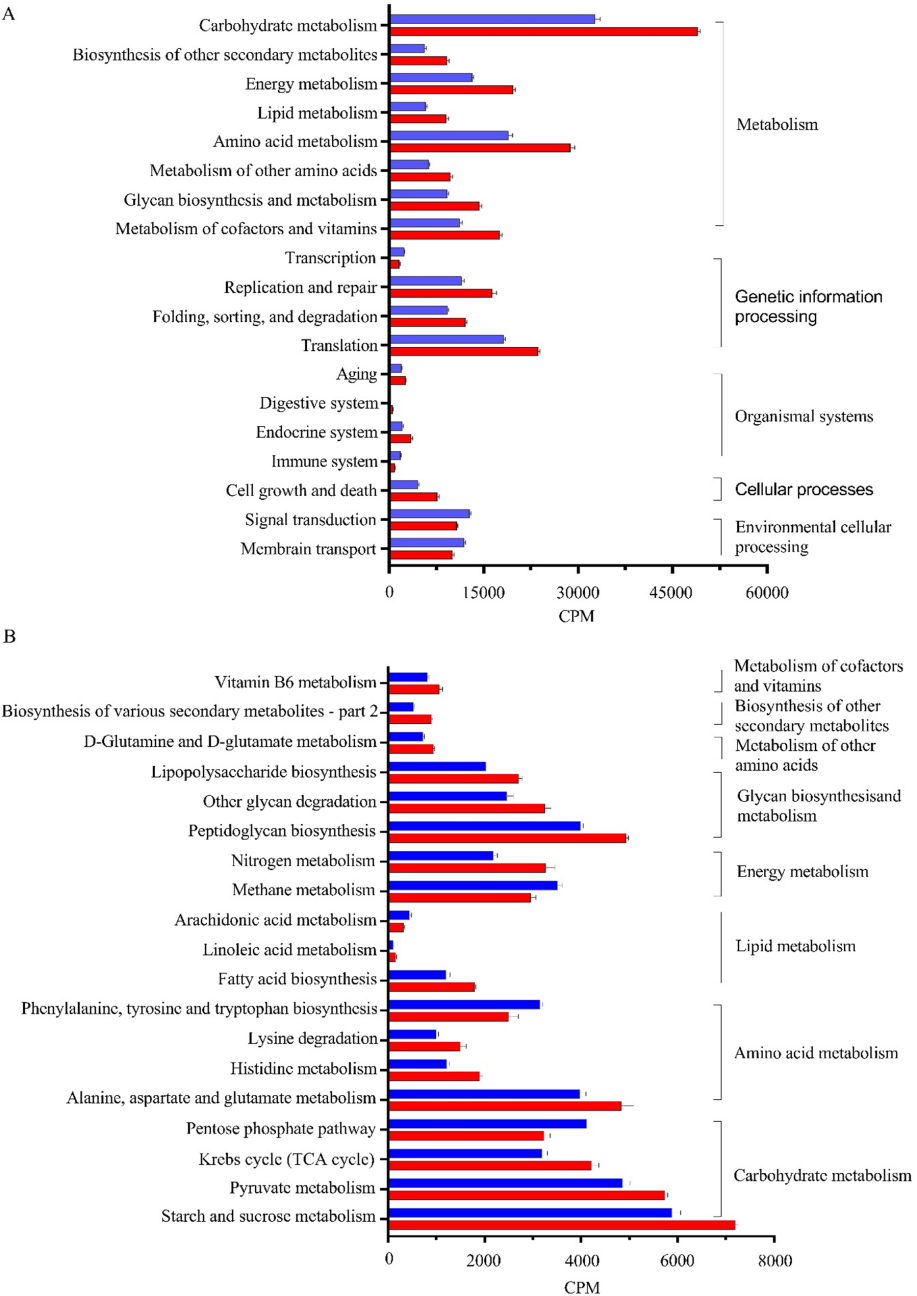
Differential Ruminal Microbial KEGG Modules Between SES and SLS Groups (*P* < 0.05). (**A**) Differentially enriched microbial KEGG modules at the second level of classification, highlighting functional variations between the SES and SLS groups. (**B**) Differential microbial KEGG pathways within the metabolism category at the third level of classification, highlighting the metabolic functional shifts in response to different sorting behavior. Red represents the SES group, while blue represents the SLS group. CPM = counts per million reads. *n* = 6

For KEGG modules, a total of 341 endogenous third-level pathways were identified as microbial metabolic pathways in the rumen of SES and SLS groups. These pathways belonged to five first-level categories, including metabolism (62.12 ± 0.84%), genetic information processing (18.82 ± 0.74%), environmental information processing (8.62 ± 0.30%), cellular processes (7.25 ± 0.04%), and organismal system (3.19 ± 0.23%) (Fig. S2). At the second level of classification, 33 categories were detected in both groups. Among them, the abundance of carbohydrate metabolism, biosynthesis of other secondary metabolites, energy metabolism, lipid metabolism, amino acid metabolism, metabolism of other amino acids, glycan biosynthesis and metabolism, metabolism of cofactors and vitamins, replication and repair, folding, sorting, and degradation, translation, aging, digestive system, endocrine system, and cell growth and death was increased in SES group (*P* < 0.05; Fig. 3A). Conversely, the abundance of transcription, immune system, signal transduction, and membrane transport was decreased in the SES group (*P* < 0.05; Fig. 3A).

To further investigate the differential metabolic categories identified within the metabolism section of the second-level KEGG classification, we analyzed the KEGG modules at the third level to pinpoint the specific metabolic pathways contributing to these differences. Only pathways under the third-level metabolism category were screened. The results revealed that four metabolic pathways related to carbohydrate metabolism were enriched. Among these, the abundance of starch and sucrose metabolism, the citrate cycle (TCA cycle), and pyruvate metabolism were enriched in the SES group, whereas the abundance of the pentose phosphate pathway was downregulated in the SES group (*P* < 0.05; Fig. 3B). For amino acid metabolism, the abundance of histidine metabolism, lysine degradation, and alanine, aspartate and glutamate metabolism was enriched in the SES group, and the abundance of phenylalanine, tyrosine and tryptophan biosynthesis was enriched in the SLS group (*P* < 0.05; Fig. 3B). In the case of lipid metabolism, the abundance of fatty acid biosynthesis and linoleic acid metabolism was enriched in the SES group, while the abundance of arachidonic acid metabolism was enriched in the SLS group (*P* < 0.05; Fig. 3B). Regarding energy metabolism, the abundance of nitrogen metabolism was elevated, and the abundance of methane metabolism was lowered in the SES group (*P* < 0.05; Fig. 3B). In glycan biosynthesis and metabolism, the abundance of lipopolysaccharide biosynthesis, peptidoglycan biosynthesis, and other glycan degradation pathways was enriched in the SES group (*P* < 0.05; Fig. 3B). Additionally, the SES group demonstrated increased abundance in pathways related to the metabolism of other amino acids (D-glutamine and D-glutamate metabolism), biosynthesis of other secondary metabolites (biosynthesis of various secondary metabolites - part 2), and metabolism of cofactors and vitamins (vitamin B6 metabolism) (*P* < 0.05; Fig. 3B).

### Functional profiling of carbohydrate metabolism

In the rumen, structural and nonstructural carbohydrates are primarily degraded into simple sugars by rumen microbiota, which are then converted into VFAs through pyruvate metabolism or oxidized via the Krebs cycle to generate more energy, with methane produced as a byproduct. To clarify the effects of excessive sorting for fine particles on carbohydrate metabolism and microbial gene abundance shifts in the SES and SLS groups, an analysis of KO genes encoding enzymes was conducted. A total of 4,175 KO genes encoding enzymes were identified in the rumen. After filtration, we obtained 1,441 genes with an abundance of more than 5 CPM (counts per million reads). Among these, 187 enzymes were matched to the KEGG database as being involved in carbohydrate metabolism. We only screened KO enzymes of carbohydrate metabolism.

**Fig. 4:**
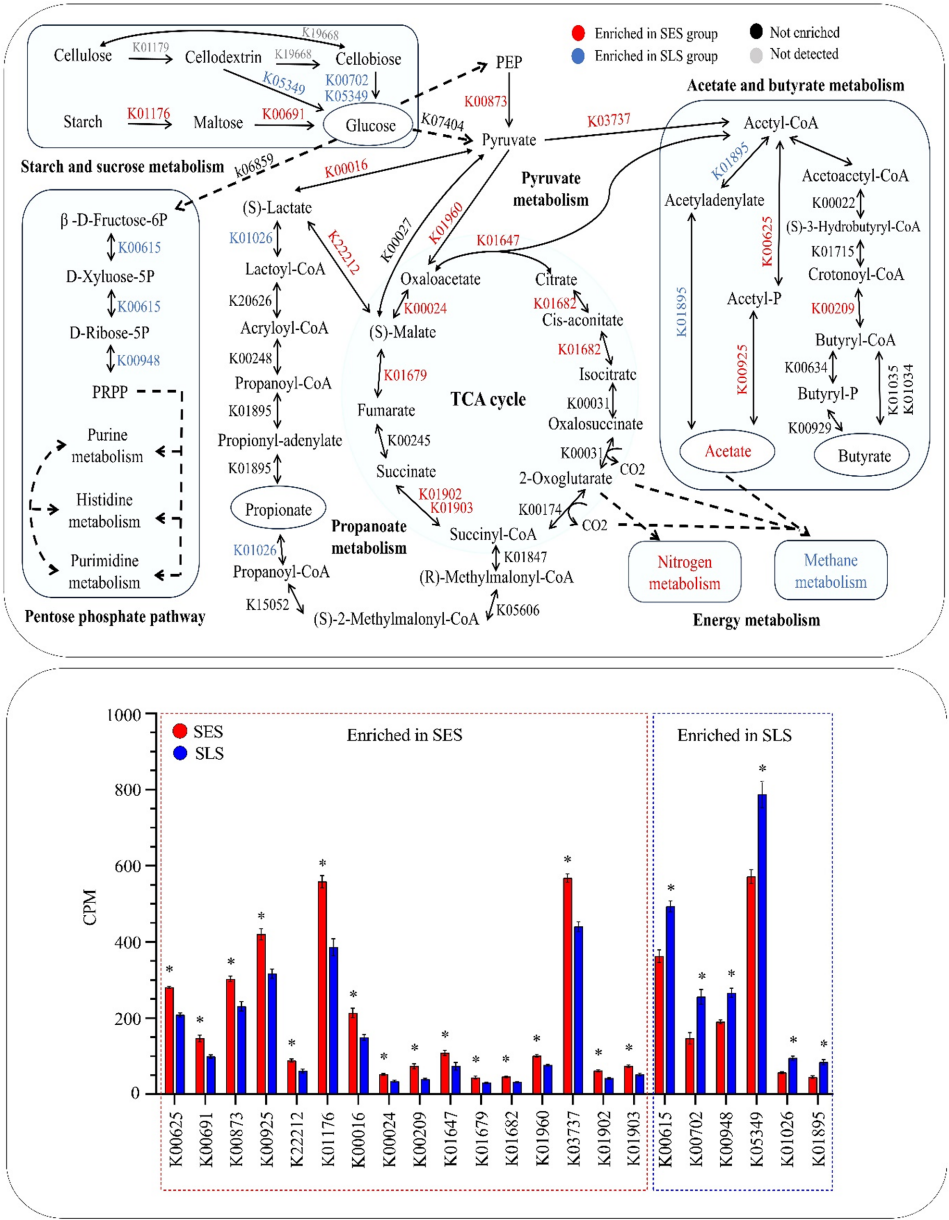
Schematic representation of changes in KO genes encoding enzymes involved in carbohydrate metabolism between SES and SLS groups. Red and blue indicate KO genes with significantly increased or decreased abundance in the SES group, respectively. Black denotes genes with no significant enrichment, and gray indicates undetected genes. PEP = phosphoenolypyruvic acid; PRPP = phosphoribosyl pyrophosphate. CPM = counts per million reads *P < 0.05; *n* = 6

Statistical analysis revealed that 22 KO gene-encoding enzymes were significantly enriched (*P* < 0.05; Fig. 4) in response to different sorting behavior. Among these, 16 genes were enriched in the SES group, while 6 genes were upregulated in the SLS group. In the starch and sucrose metabolism pathway, the SES group exhibited upregulation of gene K01176 encoding α-amylase (amyA; EC 3.2.1.1) and gene K00691 encoding maltose phosphorylase (mapA; EC 2.4.1.8), whereas gene K05349 encoding beta-glucosidase (bglX; EC 3.2.1.21) and gene K00702 encoding cellobiose phosphorylase (EC 2.4.1.20) were downregulated. Within pyruvate metabolism, the SES group showed upregulation of gene K00873 encoding pyruvate kinase (PK; EC 2.7.1.40), gene K01960 encoding pyruvate carboxylase subunit B (pycB; EC 6.4.1.1), gene K00016 encoding L-lactate dehydrogenase (LDH; EC 1.1.1.27), gene K22212 encoding malolactic enzyme (mleA; EC 4.1.1.101), and gene K03737 encoding pyruvate-ferredoxin/flavodoxin oxidoreductase (por; EC 1.2.7.1). In the TCA cycle, the SES group demonstrated upregulation of gene K00024 encoding malate dehydrogenase (mdh; EC 1.1.1.37), gene K01679 encoding fumarate hydratase class II (FH; EC 4.2.1.2), gene K01902 encoding succinyl-CoA synthetase alpha subunit (sucD; EC 6.2.1.5), gene K01903 encoding succinyl-CoA synthetase beta subunit (sucC; EC 6.2.1.5), gene K01682 encoding aconitate hydratase (acnB; EC 4.2.1.3), and gene K01647 encoding citrate synthase (CS; EC 2.3.3.1). In contrast, within propanoate metabolism, gene K01026 encoding propionate CoA-transferase (pct; EC 2.8.3.1) was enriched in the SLS group. For acetate and butyrate metabolism, the SES group exhibited downregulation of gene K01895 encoding acetyl-CoA synthetase (acs; EC 6.2.1.1), while gene K00625 encoding phosphate acetyltransferase (pta; EC 2.3.1.8), gene K00925 encoding acetate kinase (ackA; EC 2.7.2.1), and gene K00209 encoding enoyl-[acyl-carrier protein] reductase (fabV; EC 1.3.1.44) were upregulated. Additionally, the SES group downregulated KO enzymes in the pentose phosphate pathway, including gene K00615 encoding transketolase (tktA; EC 2.2.1.1) and gene K00948 encoding ribose-phosphate pyrophosphokinase (PRPS; EC 2.7.6.1). These findings highlight the differential regulation of key metabolic pathways in response to sorting behavior, providing insights into the functional shifts in microbial carbohydrate metabolism.

### Changes in the cazyme profiles in response to different sorting behavior

**Fig. 5:**
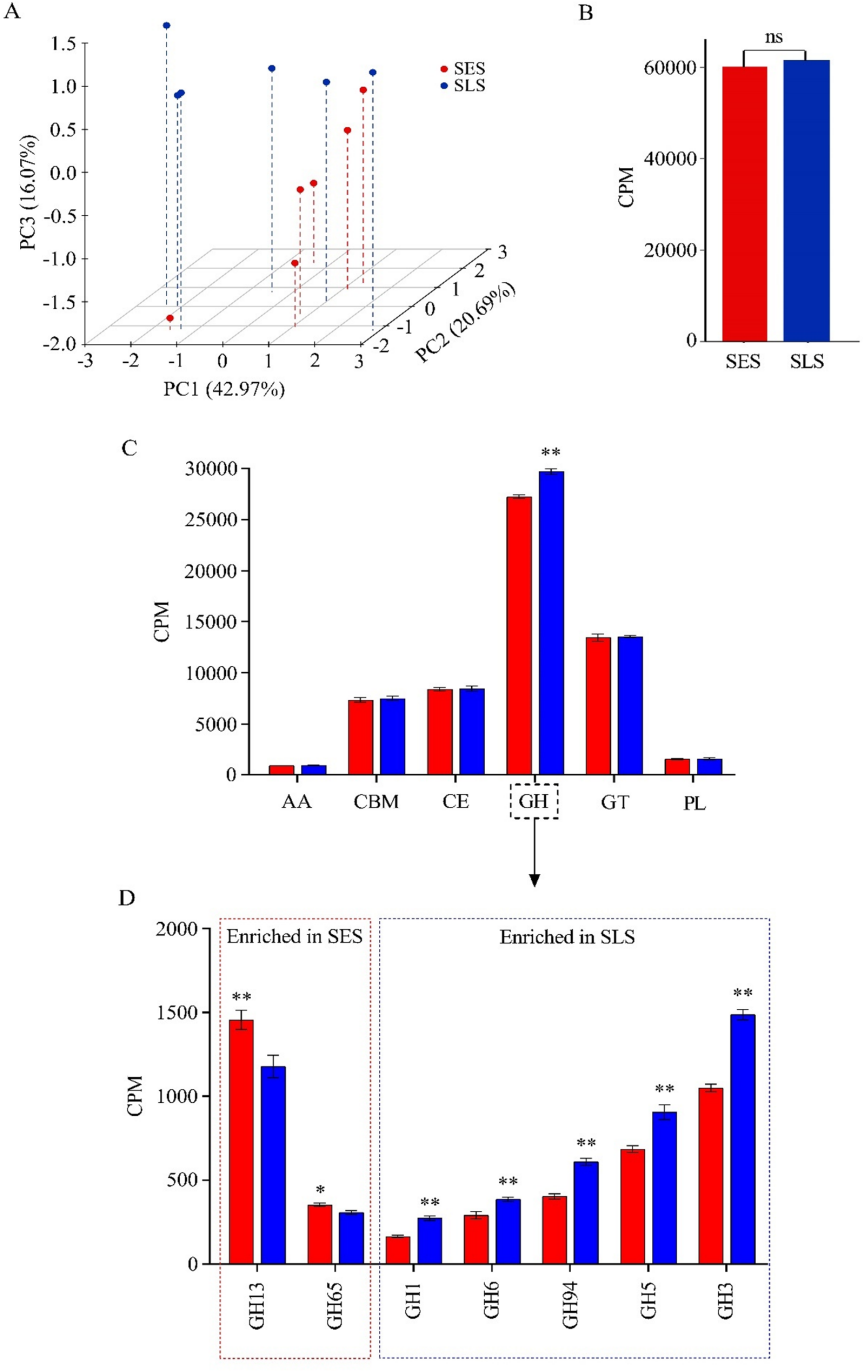
Changes in ruminal microbial CAZyme genes between SES and SLS groups. (**A**) Principal coordinate analysis of CAZyme family profiles. (**B**) Comparison of total microbial CAZyme gene abundance in the rumen of SES and SLS groups. (**C**) Differences in the abundance of CAZyme gene families, including auxiliary activities (AA), carbohydrate-binding modules (CBM), carbohydrate esterases (CE), glycoside hydrolases (GH), glycosyltransferase (GT), and polysaccharide lyases (PL), between SES and SLS groups. (**D**) Differential abundance of microbial GH family genes between SES and SLS groups (*P* < 0.05). Red represents the SES group, while blue represents the SLS group. CPM = counts per million reads *P < 0.05; *n* = 6

For CAZyme profiles, a total of 405 unique genes encoding CAZymes were identified, including 16 auxiliary activities (AAs), 93 carbohydrate-binding modules (CBMs), 18 carbohydrate esterases (CEs), 155 glycoside hydrolases (GHs), 89 glycosyltransferases (GTs), and 34 polysaccharide lyases (PLs). In general, the SES group had a lower abundance of these six classes than the SLS group (Fig. 5C). Principal coordinate analysis (PCA) of CAZyme family profiles revealed a clear separation between the two groups (Fig. 5A**).** Additionally, statistical analysis of the total abundance of CAZyme profiles was comparable between groups (Fig. 5B). However, among CAZyme classes, GHs was significantly lower in the SES group compared to the SLS group (*P* = 0.002; Fig. 5C). These findings prompted us to screen for genes encoding CAZyme families within the GHs class. After analysis, we found that starch-degrading enzymes (GH13 and GH65) were upregulated, and cellulose-degrading enzymes (GH1, GH6, GH94, GH5, and GH3) were downregulated in the SES group compared to the SLS group (*P* = 0.002; Fig. 5D). These differential CAZyme families were primarily responsible for the segregation of the SES and SLS groups observed in the PCA analysis.

### Correlation of rumen microbiota and rumen fermentation parameters

**Fig. 6:**
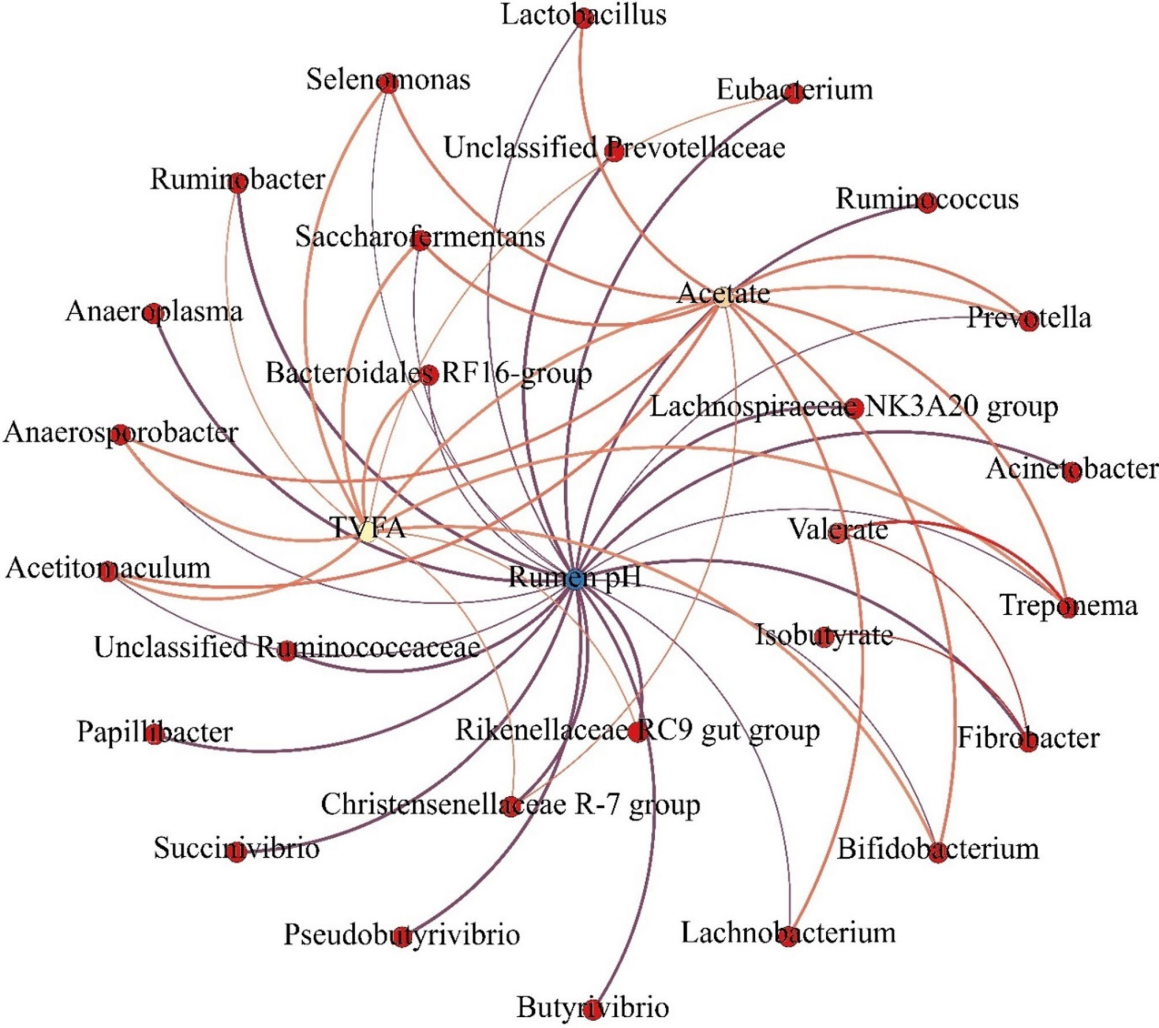
Schematic correlation networks between differential rumen bacterial genera and rumen fermentation parameters based on Spearman’s correlation coefficients (|r| >0.6, *P* < 0.05). Blue edges represent correlations network of differential genera with rumen pH, red edges represent correlations network of differential genera with VFAs, and edge thickness indicates the strength of the correlation, with thicker edges indicating stronger positive correlations and thinner edges indicating negative correlations

To investigate the intricate relationship between rumen microbiota and fermentation dynamics under varying feed sorting patterns, a correlation analysis was performed using the differential relative abundance of bacterial genera, rumen pH, and VFA concentrations. The resulting correlation network comprised 30 nodes and 50 edges (Fig. 6), including 33 positive and 17 negative correlations (|r| >0.6, *P* < 0.05). The findings revealed that the majority of ruminal bacteria in the SES group exhibited positive correlations with acetate and TVFA concentrations and negative correlations with ruminal pH. For instance, the relative abundances of *Prevotella*,* Lactobacillus*,* Bifidobacterium*,* Selenomonas*,* Treponema*,* Lachnobacterium*,* Saccharofermentans*, and *Acetitomaculum* were positively correlated with acetate levels and negatively correlated with ruminal pH. Conversely, in the SLS group, several bacterial genera, including *Fibrobacter*,* Ruminobacter*,* Pseudobutyrivibrio*,* Butyrivibrio*,* Papillibacter*,* Lachnospiraceae NK3A20 group*,* Unclassified Prevotellaceae*,* Christensenellaceae R_7 group*,* Eubacterium*,* Unclassified Ruminococcaceae*,* Rikenellaceae RC9 gut group*,* Acinetobacter*,* Succinivibrio*,* Ruminococcus*, and *Anaeroplasma*, demonstrated positive correlations with ruminal pH (Fig. 6).

## Discussion

### Variations in feed sorting behavior distinctly altered rumen fermentation profiles and modified fiber digestibility in SES and SLS cows

Feed sorting is a well-recognized but undesirable behavior exhibited by dairy cows, as it can significantly disrupt rumen fermentation patterns and metabolic processes. Previous studies have consistently demonstrated that dairy cows tend to sort TMR in favor of shorter particles [3, 11]. However, the extent of this behavior varies considerably among individual cows and across farms, influenced by factors such as management practices and environmental conditions [4, 12]. In the present study, the same TMR offered resulted in different degrees of feed sorting among the experimental cows, and 12 cows were selected and classified into two groups based on their degree of sorting behavior for fine particles. The analysis of feed sorting indices of the two groups over the course of the experimental period showed that sorting time had no significant effect on the sorting behavior of each group, implying that all cows maintained consistent feed sorting behavior over the course of the experimental period. The SES group exhibited a pronounced preference for fine particles, rejecting long, medium, and short particles to a greater extent than the SLS group. This result suggests substantial variations in the intake of structural and non-structural carbohydrates between the two groups.

The impact of feed sorting on rumen pH is intricately linked to the dynamics of rumen fermentation processes. Excessive sorting for fine particles compromises the consistency of nutrient intake, often elevating the intake of rapidly fermentable carbohydrates while reducing the intake of effective fiber. This imbalance disrupts the stability of rumen fermentation, resulting in heightened production of VFA and lactic acid, which subsequently declines rumen pH [13–15]. In this study, the SES group exhibited significantly higher concentrations of TVFA and acetate while recording lower rumen pH values compared to the SLS group, providing clear evidence of the strong link between excessive sorting for fine particles and rumen fermentation dynamics. Regarding the SARA challenge, previous research has demonstrated that sorting TMR against long particles increased the susceptibility to SARA [3, 16]. Overall, SARA challenge occurs when the ruminal pH remains between 5.2 and 6 for prolonged times [17]. In the current study, the SES group recorded lower rumen pH values than the SLS group (pH = 6.14 vs. 6.46), indicating that excessive sorting for fine particles may exacerbate the decline in ruminal pH compared to slight sorting. This further highlights the potential for increased susceptibility to SARA if the sorting behavior continues to be more extreme for fine particles, as the reduction in rumen pH can induce alterations in rumen microbiota structure, shifting fermentation towards increased organic acid production, and further inducing pH depression in the rumen.

Another important finding in this study was that the SES group exhibited lower apparent digestibility of NDF and ADF compared to the SLS group. This result highlights the negative effects of excessive sorting behavior on rumen function. Excessive sorting for finer particles often leads to reduced intake of fiber-rich particles, which are essential for maintaining optimal rumen function and microbial activity, especially cellulolytic bacteria [18]. The reduced fiber digestibility observed in the SES group may be attributed to the decreased intake of peNDF, which is critical for maintaining rumen pH and cellulolytic bacteria structure and function in the rumen. Additionally, the nutrient retention time plays an important role in nutrient digestibility. The SES group is theoretically expected to have less retention time and lower nutrient digestibility compared to the SLS group due to increasing finer particle intake [19]. However, the lack of difference in DM, OM, EE, and CP digestibility between SES and SLS groups suggests that retention time is unlikely to be the primary reason for these results. Instead, the suggested negative impact of finer particles on the rumen microbiota appears to be the primary factor contributing to the reduced fiber digestibility observed in the SES group.

### Variations in bacterial communities were primarily responsible for the differences in fermentation patterns and fiber digestibility in the rumen between SES and SLS groups

In the current study, excessive sorting for fine particles had no significant effect on ruminal archaea, eukaryota, or viruses. However, the relative abundance of bacteria decreased in the SES group compared to the SLS group, prompting us to further investigate ruminal bacterial communities. The PCoA analysis of ruminal bacterial profiles revealed clear segregation between the two groups, indicating that feed sorting behavior significantly influences the structural composition of the rumen microbiota. Alpha diversity metrics further supported this observation, with the SES group exhibiting significantly lower bacterial richness compared to the SLS group, as evidenced by the observed species and Chao1 indices. However, no significant differences were observed in the Shannon and Simpson indices, suggesting that while species richness was reduced in the SES group, the evenness of species distribution remained relatively unaffected.

At the phylum level, *Bacteroidota* and *Firmicutes* dominated the rumen microbiota in both groups, consistent with previous studies that identify these phyla as key players in rumen fermentation [20, 21]. However, the relative abundance of *Bacteroidota*, *Bacillota_C*, and *Actinomycetota* increased, while the relative abundance of *Bacillota_A*, *Fibrobacterota*, and *Pseudomonadota* decreased in the SES group compared to the SLS group. These shifts suggest that excessive sorting for fine particles may favor the proliferation of certain bacterial taxa more specialized in fermenting easily fermented substrates and reduce the population of fiber-degrading bacteria, potentially impacting the overall functional capacity of the rumen microbiome. At the genus level, consistent with many previous studies [22, 23], *Prevotella* emerged as the most abundant genus in both groups, with a higher relative abundance in the SES group. Species such as *Prevotella albensis*, *Prevotella bryantii*, *Prevotella ruminicola*, and *Prevotella brevis* were enriched in the SES group, consistent with their roles in starch fermentation and carbohydrate metabolism [24–26]. This study further revealed that the higher relative abundance of *Prevotella* in the SES group was positively correlated with acetate and TVFA concentrations but negatively correlated with ruminal pH. These findings confirmed that while *Prevotella* is essential for energy metabolism, its higher proliferations in the rumen can lead to excessive VFA production, increasing the risk of low rumen pH as documented in previous studies [27, 28]. Similarly, other starch-fermenting genera, including *Lactobacillus* (*Lactobacillus acidophilus*) [29], *Bifidobacterium* (*Bifidobacterium adolescentis* and *Bifidobacterium merycicum*) [30], *Selenomonas* (*Selenomonas ruminantium*) [31], *Saccharofermentans* (*Saccharofermentans acetigenes*) [32], *Treponema* (*Treponema bryantii*) [33], *Lachnobacterium* (*Lachnobacterium bovis*) [34], and *Acetitomaculum* (*Acetitomaculum ruminis*) [35], were also more abundant in the SES group. These genera are known for their roles in carbohydrate metabolism. Specifically, *Lactobacillus acidophilus*, *Bifidobacterium adolescentis*, and *Selenomonas ruminantium* are recognized for their ability to ferment starch and release lactic acid in the rumen [36], exacerbating the risk of low rumen pH. *Saccharofermentans*, which ferments glucose to produce acetate and lactate, has been associated with laminitis in previous studies [37]. Additionally, *Lachnobacterium* has been reported to increase in abundance during SARA challenges [38, 39]. The correlation analysis in this study reinforced these findings, showing that the enriched genera in the SES group were positively correlated with VFA concentrations and negatively correlated with ruminal pH. These results highlight the critical role of starch-fermenting bacteria in driving VFA production and acid accumulation in the rumen in response to excessive sorting for fine particles.

Conversely, fiber-degrading genera such as *Fibrobacter* (*Fibrobacter succinogenes*), Ruminococcus (Ruminococcus albus and *Ruminococcus flavefaciens*), *Butyrivibrio* (*Butyrivibrio fibrisolvens* and Butyrivibrio proteoclasticus), Pseudobutyrivibrio (Pseudobutyrivibrio xylanivorans), Ruminobacter (*Ruminobacter amylophilus*), and *Papillibacter* (*Papillibacter cinnamivorans*) were less abundant in the SES group. These genera are critical for the degradation of structural carbohydrates such as cellulose and hemicellulose [40], and their suppression likely contributed to the lower apparent digestibility of NDF and ADF observed in the SES group. The reduced abundance of *Christensenellaceae R_7 group*, *Lachnospiraceae NK3A20 group*, *Unclassified Ruminococcaceae*, and *Unclassified Prevotellaceae*, which are also associated with fiber degradation and rumen micro-ecological stability [20, 41, 42], further highlights the negative impact of excessive sorting on fiber digestion and rumen function. These findings collectively suggest that excessive sorting for fine particles may shift the rumen microbiota toward a community more specialized in starch and sugar metabolism, at the expense of fiber-degrading taxa. Regarding concerns about pathogenic bacteria, no increase in opportunistic or pathogenic taxa was detected in the SES group. Notably, the relative abundance of *Acinetobacter*, including *Acinetobacter baumannii*, was significantly decreased in SES cows. This decline likely reflects the lower ruminal pH induced by excessive sorting for fine particles and increased starch fermentation in the rumen. Recent evidence has highlighted the sensitivity of *Acinetobacter baumannii* to acidic conditions, demonstrating that low pH environments inhibit its growth and biofilm formation [43]. Therefore, rather than promoting pathogenic bacteria, the acidotic ruminal environment in SES group appears to exert selective pressure that suppresses pH-sensitive opportunistic taxa.

### Variations in microbial metabolic pathways, KO enzymes, and CAZymes in response to different sorting behavior were primarily responsible for changes in the carbohydrate metabolism and VFA production in the rumen

The functional profiling of microbial genes further elucidates the metabolic shifts associated with feed sorting behavior. The functional analysis of the rumen microbiome revealed significant metabolic differences between the SES and SLS groups. The SES group exhibited increased relative abundances of pathways related to carbohydrate metabolism, including starch and sucrose metabolism, pyruvate metabolism, and the TCA cycle. This was accompanied by the upregulation of key KO genes encoding enzymes involved in these pathways. In starch and sucrose metabolism, key enzymes such as K01176 (amyA) and K00691 (mapA) were upregulated, whereas K05349 (bglX) and K00702 (EC 2.4.1.20) were downregulated in the SES group. The amyA gene encodes α-amylase, an enzyme with extracellular localization primarily produced by certain *Prevotella* and *Lactobacillus* species [24, 44], while the mapA gene encodes maltose phosphorylase, also with extracellular localization, and is primarily produced by *Lactobacillus* species [44]. Therefore, we deduce that when cows are excessively sorted TMR for fine particles, the proliferation of *Prevotella* and *Lactobacillus* increases rapidly, which enriches the amyA and mapA in the rumen, accelerating the starch degradation in the rumen. In addition, the bglX gene encodes beta-glucosidase, and the EC 2.4.1.20 gene encodes cellobiose phosphorylase, both of which are involved in cellulose and hemicellulose degradation. These enzymes are typically found in *Fibrobacter* and *Ruminococcus*, two main fiber degradation genera in the rumen [45, 46]. The lower abundance of these genera in the SES group likely explains the downregulation of bglX and EC 2.4.1.20 enzymes, leading to decreased efficiency in cellulose and hemicellulose breakdown in the rumen. This aligns with the observed lower NDF and ADF digestibility in the SES group.

The simple sugars in the rumen are fermented into VFAs by the majority of rumen microbiota, with pyruvate metabolism serving as a central pathway in this process. In pyruvate metabolism, enzymes such as K00873 (PK), K00016 (LDH), and K01960 (pycB) were upregulated in the SES group. The PK catalyzes the final step of glycolysis, converting phosphoenolpyruvate (PEP) to pyruvate while generating ATP. The upregulation of PK suggests an increased glycolytic flux in the SES group, likely driven by the higher availability of fermentable carbohydrates. This enhanced glycolysis provides more pyruvate as a substrate for downstream metabolic pathways, such as the TCA cycle or lactate production. The LDH converts pyruvate to lactate, regenerating NAD ^+^ for continued glycolysis [47]. The LDH can also catalyze lactate back into pyruvate for generating more acetate and butyrate in certain conditions [48]. The upregulation of LDH indicates a shift toward lactate production and utilization, which is common in carbohydrate-rich diets. This metabolic adaptation helps maintain energy production in the rumen but may also lead to lactate accumulation, potentially leading to lower rumen pH. However, previous studies indicated a significant increase in the conversion of lactate into acetate and butyrate in the rumen during SARA challenges [49]. The upregulation of K22212 (mleA) in the SES group further indicates a metabolic shift toward lactate production, in which mleA facilitates lactate production from malate. In addition, the downregulation of K01026 (pct), an enzyme involved in converting lactate to propionate, suggests a potential limitation in lactate utilization into propionate. Simultaneously, the upregulation of K01960 (pycB), which catalyzes the conversion of pyruvate to oxaloacetate, along with other TCA cycle enzymes such as K01647 (CS), K00024 (mdh), and K01679 (FH), indicates that the rumen microbiota in the SES group is prioritizing oxidative metabolism to meet energy demands. In terms of rumen microbiota, *Selenomonas* [50] and *Bifidobacterium* [51] are known to produce PK, while *Lactobacillus* is known to produce mleA [52]. Additionally, *Lactobacillus* [53] and *Selenomonas* [54] have the ability to encode LDH. While *Selenomonas* is primarily recognized for its role in fermenting lactate to propionate, it is also capable of producing lactate under certain conditions or even converting lactate into pyruvate for more acetate and butyrate production. Previous studies have shown that during SARA challenges, *Selenomonas* shifts its metabolic activity, utilizing more lactate to produce acetate and butyrate rather than propionate, resulting in lower propionate levels [49]. We deduce that a higher abundance of LDH and mleA from *Lactobacillus* increases the lactic acid release in the rumen of the SES group, and at least part of the lactic acid is utilized by *Selenomonas ruminantium* to increase acetate production.

In acetate and butyrate metabolism, the SES group demonstrated an upregulation of K03737 (por), which encodes pyruvate: ferredoxin oxidoreductase, a critical enzyme that converts pyruvate into acetyl-CoA, a central intermediate in metabolic pathways, including acetate and butyrate production. This upregulation suggests a metabolic shift favoring acetyl-CoA production, which is essential for short-chain fatty acid (SCFA) synthesis. Furthermore, the SES group exhibited upregulation of K00625 (pta) and K00925 (ackA), the two main enzymes in the phosphate acetyltransferase-acetate kinase (Pta-AckA) pathway, while downregulating K01895 (acs), which encodes acetyl-CoA synthetase. This indicates that acetate production in the SES group is primarily driven by the Pta-AckA pathway, a thermodynamically favorable and energy-efficient route that catalyzes the conversion of acetyl-CoA to acetate with ATP generation. The downregulation of acs reflects a reduced reliance on acetate assimilation, emphasizing acetate excretion as a metabolic endpoint, which aligns with the observed higher acetate concentration in the rumen of the SES group. These results are further supported by the increased abundance of acetate-producing bacteria, including *Prevotella*, *Selenomonas*, *Treponema*, *Saccharofermentans*, and *Acetitomaculum*, which collectively suggested contributing to the elevated acetate levels observed in the SES group [55–61]. Most of these genera likely produce acetate via the Pta-AckA pathway, a typical metabolic route in acetogenesis. However, *Selenomonas ruminantium* appears to utilize an atypical pathway or contributes to acetate production by elevating acetyl-CoA levels, due to the absence of ackA and pta activities, as reported in previous studies [62]. Additionally, the SES group showed an upregulation of K00209 (fabV), which encodes enoyl-ACP reductase, a critical enzyme involved in fatty acid biosynthesis and butyrate production [63, 64]. In this study, butyrate levels were comparable between the SES and SLS groups, suggesting an increased demand for fatty acid biosynthesis rather than butyrate production. This suggests that microbiota enhanced fatty acid production, potentially to support membrane synthesis or energy storage, as part of an adaptive response to maintain cellular homeostasis or cope with environmental stresses in the rumen in response to excessive sorting for fine particles.

Regarding the pentose phosphate pathway, the SES group downregulated K00615 (tktA) and K00948 (PRPS), the two enzymes essential for producing NADPH and ribose-5-phosphate, critical for nucleotide and amino acid biosynthesis. This metabolic reallocation favored energy production and carbohydrate metabolism, supported by the enrichment of glycolytic and TCA cycle pathways and increased amino acid degradation (e.g., histidine metabolism and lysine degradation), while biosynthetic pathways (e.g., phenylalanine, tyrosine, and tryptophan biosynthesis) were downregulated. Consistent with previous findings in high-grain diets [10], the SES group also exhibited upregulated nitrogen metabolism and downregulated methane metabolism. The enhanced nitrogen metabolism can be attributed to increased oxoglutarate formation in the TCA cycle, facilitated by the upregulation of genes such as K01647 (CS) and K01682 (acnB), which promote the conversion of oxoglutarate to glutamate and subsequently to other amino acids. Furthermore, the downregulation of methane metabolism can be attributed to the higher relative abundance of acetogenic bacteria in the rumen of the SES group, such as *Acetitomaculum*, which utilize CO_2_ and H_2_ to produce acetate, thereby reducing the substrates available for methanogenesis [65].

The results of the CAZyme profiles align closely with the findings of KO enzymes, highlighting significant differences in carbohydrate metabolism between groups. In the SES group, CAZyme families related to starch metabolism, such as GH13 and GH65, were upregulated. These enzymes are primarily produced by certain amylolytic bacteria in the rumen, suggesting that the higher relative abundance of starch-degrading bacteria in the rumen, including *Prevotella* and *Lactobacillus*, contributed to the increased production of these enzymes. Conversely, the SES group exhibited downregulation of CAZyme families associated with cellulose, hemicellulose, and oligosaccharide metabolism, including GH1, GH6, GH94, GH5, and GH3. These enzymes are predominantly produced by cellulolytic bacteria such as *Ruminococcus*, *Fibrobacter*, *Butyrivibrio*, and *Pseudobutyrivibrio* [66, 67]. The lower relative abundance of these cellulolytic bacteria in the rumen of the SES group likely explains the reduced abundance of these CAZyme families. This reduction in cellulolytic enzyme activity may be a key factor contributing to the lower digestibility of ADF and NDF observed in the SES group.

## Conclusions

**Fig. 7:**
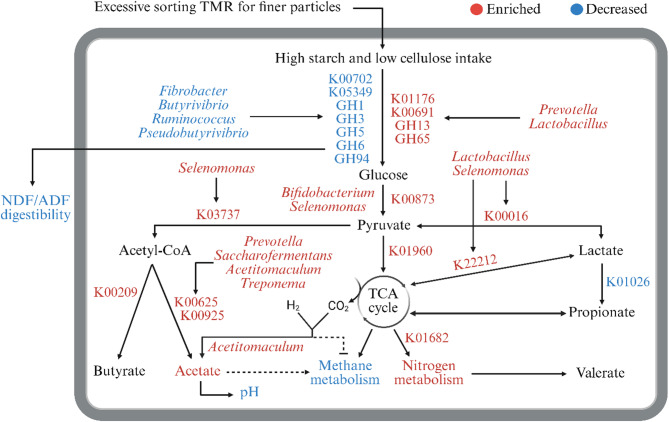
Schematic flowchart illustrating changes in rumen microbiome and gene functions involved in carbohydrate metabolism in response to excessive sorting TMR for finer particles in dairy cows. In this flowchart, excessive sorting for fine particles increased the relative abundance of ruminal bacterial taxa more specialized in starch metabolism (*Prevotella*,* Lactobacillus*,* Selenomonas*,* Bifidobacterium*,* Treponema*,* saccharofermentans*,* Acetitomaculum*) at the expense of fiber-degrading taxa (*Fibrobacter*,* Ruminococcus*,* Butyrivibrio*,* Pseudobutyrivibrio*). This microbial shift was associated with the upregulation of key enzymes involved in starch metabolism, glycolysis, and acetate production, while fiber-degrading enzymes were downregulated, ultimately reducing the digestibility of ADF and NDF. These changes collectively contributed to a reduction in rumen pH. Red indicates enriched abundance, and blue indicates reduced abundance

The key findings of this study are summarized in Fig. 7. Our findings highlight the detrimental implications of feed sorting on rumen function and fiber digestibility in dairy cows. Excessive sorting for fine particles in TMR increased the relative abundance of bacterial taxa more specialized in starch metabolism (*Prevotella*, *Lactobacillus*, *Selenomonas*, *Bifidobacterium*, *Acetitomaculum*) at the expense of fiber-degrading taxa (*Fibrobacter*, *Ruminococcus*, *Butyrivibrio*, *Pseudobutyrivibrio*). These changes were accompanied by upregulation of key enzymes involved in starch metabolism, glycolysis, acetate production, and fatty acid biosynthesis, alongside downregulation of fiber-degrading enzymes. This resulted in lower rumen pH, increased TVFA and acetate concentrations, and reduced digestibility of ADF and NDF. Overall, our findings indicate that the rumen microbiota and its functionality are influenced not only by imbalances in dietary composition but also by how cows consume a well-balanced TMR. To mitigate the adverse effects of feed sorting on rumen health and digestibility, we recommend implementing early detection tools, such as electronic feeders and machine vision systems, to monitor individual feeding behavior. Detection of sorting should be followed by targeted mitigation strategies, including optimizing forage particle size and enhancing feed bunk management. Further research is warranted to explore the implications of feed sorting on host metabolism and overall cow health.

## Methods

### Animals, housing, and experimental design

Twenty-four multiparous Holstein cows in mid-lactation, with an average body weight of 581.59 kg (range: 512.39–631.24 kg), and an average parity of 4.00 (range: 3–5), were initially selected for this study. The cows were housed in a tethered barn and had *ad libitum* access to feed and water. The experiment was performed over 28 days, including a 7-day pre-trial period followed by a 21-day main trial period. The cows were fed a TMR with a 50:50 forage-to-concentrate ratio, formulated in accordance with the Chinese dairy cow feeding standard requirements [68]. The composition and nutritional levels of the TMR are detailed in Table 4. Fresh TMR was prepared three times daily at 7:30 a.m., 1:00 p.m., and 6:00 p.m. and was distributed in equal portions using an automatic feeding system. The fresh TMR was provided in quantities that allowed for a minimum of 10% feed refusals. Feed bunks were emptied and thoroughly cleaned each day prior to the morning feeding. Fresh TMR samples were obtained at the delivery time and within minutes of delivery from five distinct points along the feed bunk to ensure accurate representation. Leftover samples from each cow were collected individually three hours post-feeding, following thorough mixing of each cow’s leftovers. At each sampling time, the collected samples were promptly separated into four particle size fractions using a 3-sieve and bottom-pan Penn State Particle Separator (PSPS, model C24682N, Nasco, Fort Atkinson, WI): long particles (> 19 mm), medium particles (8–19 mm), short particles (1.18–8 mm), and fine particles (< 1.18 mm), following the methodology described by [69]. The degree of feed sorting for each fraction was assessed, and feed sorting indices were calculated by determining the ratio of actual intake to expected intake for the particles retained on each layer of the PSPS [70]. The anticipated intake for each fraction was calculated by multiplying the dry matter intake (DMI) of the total diet by the dry matter (DM) percentage of that fraction present in the delivered TMR.

**Table 4:** Ingredients and chemical composition of the experimental TMR

Item	%
Ingredients, % of DM	
Corn	19.33
Beer lees	10.03
Corn silage	28.08
Leymus chinensis	5.58
Alfalfa hay	11.47
Soybean meal	11.03
Wheat bran	2.78
DDGS	4.39
Molasses bean skin	2.83
NaCl	0.55
NaHCO_3_	0.83
Premix^1^	2.25
Mycotoxin adsorbents	0.06
CaHPO_4_	0.59
Dried yeast	0.19
Total	100.00
Nutrient composition	
DM, %	47.23
OM, %	39.81
EE, % of DM	3.91
CP, % of DM	16.44
NDF, % of DM	35.03
ADF, % of DM	18.45
Ash, % of DM	7.42
NFC^2^, % of DM	37.20
NFC/NDF	1.06

DDGS = distiller’s dried grains with solubles, EE = ether extract, CP = crude protein, NDF = neutral detergent fiber, ADF = acid detergent fiber. NFC = non-fiber carbohydrate

^1^The premix provided the following nutrients per kilogram of diet: vitamin D3 (6.0 kIU), vitamin A (21.5 kIU), vitamin K3 (5.0 mg), vitamin E (39.5 IU), Mn (64.5 mg), Cu (25.6 mg), Zn (112.9 mg), and Fe (159.3 mg)

^2^NFC (%) = 100 - [NDF (%) + CP (%) + EE (%) + ash (%)]

### Feed chemical analysis and digestibility

On days 1, 7, 14, and 21 of the main trial period, samples of the offered TMR in morning, noon, and night feeding were collected randomly from different feeding points and well mixed in polythene bags before being frozen at − 20 °C. Approximately 500 g of the mixed TMR sample was divided into four fractions using PSPS. Each fraction was weighed, placed in polythene bags, and stored at − 20 °C for downstream analysis. On days 1, 7, 14, and 21 of the main trial period, feces samples were collected from each cow’s rectum four hours after the morning feeding and immediately preserved using 20 ml of sulfuric acid 10% (vol/vol) and stored at − 20 °C for subsequent analysis. Before chemical analysis, the feces samples of each cow were well mixed, and the representative samples of TMR, feces, and PSPS fractions were taken using the quadrat sampling method. The representative samples were later analyzed to determine their chemical composition. The contents of DM (method 934.01), CP (method 976.05), EE (method 973.18), crude ash (method 942.05), and ADF (method 973.18) in TMR were assessed following the methods described in AOAC [71]. The NDF content was evaluated using the Van Soest method [72], which involved the use of heat-stable alpha-amylase and sodium sulfite. The organic matter (OM) content was calculated as the difference between DM and crude ash. The non-fiber carbohydrate (NFC) in TMR was calculated using the following equation: NFC = 100 - [NDF % + CP % + EE % + ash %]. The detailed chemical composition of the experimental TMR and the PSPS fractions are presented in Tables 4 and 5, respectively. To calculate the percentage of apparent nutrient digestibility, the following equation was used: Digestibility = [(Nutrient intake - Nutrient excretion in feces) / Nutrient intake] × 100.

**Table 5:** Detailed chemical composition of the experimental TMR fractions separated by PSPS^1^

Item	> 19 mm	8–19 mm	1.18–8 mm	< 1.18 mm
Weight, g	71.40	115.17	96.21	217.22
DM, %	46.12	47.51	49.75	51.88
OM, %	37.80	39.81	42.26	43.48
EE, % of DM	3.87	4.16	4.92	5.86
CP, % of DM	12.21	14.31	17.12	19.87
NDF, % of DM	42.72	38.16	29.99	17.65
ADF, % of DM	28.52	19.24	12.14	7.12
Ash, % of DM	8.32	7.70	7.49	8.40
NFC^2^, % of DM	32.88	35.67	40.48	48.22
NFC/NDF	0.77	0.93	1.35	2.73

PSPS = Penn state particle separator, EE = ether extract, CP = crude protein, NDF = neutral detergent fiber, ADF = acid detergent fiber. NFC = non-fiber carbohydrate

^1^A total of 500 g of TMR (as fed) was separated into four fractions for analysis

^2^NFC (%) = 100 - [NDF (%) + CP (%) + EE (%) + ash (%)]

### Rumen content sampling and VFA analysis

On the last day of the experimental period, rumen content samples from all cows were collected 3 h after morning feeding using a stomach tube into 50 ml tubes. To prevent any possible contamination from saliva, approximately 150 ml of the initially gathered rumen content was discarded before the collection of samples. Following the collection, the ruminal pH was assessed immediately using a pH meter (206-pH2; Testo AG, Germany). The ruminal content samples were then divided into two portions: the first portion was preserved in liquid nitrogen for subsequent microbial DNA extraction, while the second portion was filtered through four layers of gauze to obtain ruminal fluid. The filtered ruminal fluid samples were stored at − 20 °C for VFA determination. The concentrations of VFA, including acetate, propionate, butyrate, isobutyrate, valerate, and isovalerate, were determined using the gas chromatography technique (GC-2014B, Shimadzu, Japan; capillary column specifications: 30 m × 0.32 mm × 0.25 μm; detector temperature = 210 °C; gasification temperature = 220 °C; chromatographic column temperature = 160 °C), following the methodologies outlined by [73].

### Total DNA extraction

The microbial DNA was extracted from 0.3 g of ruminal content per sample using the E.Z.N.A.^®^ Soil DNA Kit (Omega Bio-Tek, Norcross, GA, USA). The extraction procedure was conducted in strict accordance with the standard operating protocol provided by the manufacturer. A bead-beating method was employed to break down microbial cell walls and facilitate the release of DNA. The purity and concentration of the extracted DNA were assessed using a NanoDrop spectrophotometer (NanoDrop 1000, Thermo Fisher Scientific, Madison, USA) and 1% agarose gel electrophoresis. All extracted DNA samples were subsequently stored at − 80 °C until further analysis [74].

### DNA library construction and sequencing

For each sample, 1 µg of genomic DNA was fragmented into approximately 450 bp fragments using the Covaris M220 ultrasonicator (Covaris Inc., Woburn, MA). Paired-end metagenomic libraries were subsequently constructed using the TruSeq DNA Sample Prep Kit (Illumina, San Diego, CA) and were sequenced on the Illumina HiSeq X Ten platform.

Following sequencing, low-quality reads, as well as contaminating adaptor sequences, were eliminated from the raw sequencing data using Trimmomatic [75]. To further remove host-genome contamination, the reads were aligned to the Bos taurus ARS-UCD1.2 reference genome (obtained from the National Center for Biotechnology Information) using the BWA-MEM (v0.7.17) [76]. The resulting clean reads were then processed with MEGAHIT (v1.1.1), employing the option of “--min-contig-len 500“ to assemble the data [77]. Contig abundance was then quantified using Salmon (v1.9.0) [78], run in metagenomic mode (--meta) with selective alignment enabled (--validateMappings). Mean coverage per contig was calculated using Salmon’s output. Contigs with mean coverage < 60% were subsequently discarded to ensure robust downstream analysis. Open reading frames (ORFs) were predicted from the filtered contigs using Prodigal (v2.6.3) in metagenomic mode (-p meta) [79], and the derived ORFs were clustered into a non-redundant gene catalog using CD-HIT (v4.6.7) [80], with a sequence identity threshold of 0.95 [81].

### Taxonomic classification and functional annotation

The non-redundant gene catalog was translated into amino acid sequences using EBI-TRANSEQ (https://www.ebi.ac.uk/Tools/st/emboss_transeq/) [82]. Taxonomic annotation was performed using BLASTP (v2.12.0+) against the NCBI NR database (https://www.ncbi.nlm.nih.gov/refseq/about/nonredundantproteins/), with an e-value threshold of 1e-5 and a minimum alignment length of 60 amino acids. Functional annotation was conducted using BLASTP (v2.12.0+) against the KEGG database (https://www.genome.jp/kegg/) [83]. CAZyme identification was performed using hmmscan (HMMER v3.3.2) against the dbCAN3 CAZy HMM database (http://www.cazy.org/) [84]. Taxonomic profiles were established at the domain, phylum, class, family, genus, and species levels. Features with a relative abundance > 0.1% in at least one group were retained for downstream analysis. The abundances of KEGG modules, KO genes, and CAZymes were normalized to CPM. For statistical analyses, only features with CPM > 5 in at least one group were included.

### Statistical analysis

Sample size determination using G*Power software (v 3.1.9.6) based on a t-test for dependent means (α = 0.01, power = 80%) revealed that a minimum of 6 cows in each group was required. Feed sorting behavior was identified by averaging feed sorting data of all cows across days 1, 7, 14, and 21 of the main trial period. Cows were then categorized based on their sorting indices into two groups: severe sorting (SES; *n* = 6), representing excessive sorting for fine particles, and slight sorting (SLS; *n* = 6), representing slight sorting for fine particles. Furthermore, 12 cows were excluded from the analysis due to either consistent normal sorting across all particle sizes or inconsistent sorting for fine particles throughout the experimental period.

Statistical analysis of feed sorting indices was performed using the linear mixed model (MIXED) procedure in SPSS software (IBM SPSS v. 24; IBM Corp., Armonk, NY, USA), with the model: Yij = µ + A_i_ + P_j_ + (AP)_ij_ + e_ij_, where µ is the overall mean, A_i_ is the fixed effect of sorting group (i = 1–2), P_j_ is the fixed effect of day (j = 1–4), (AP)_ij_ is the fixed effect of the sorting group by day interaction, and eij is the random residual error. Furthermore, the independent t-tests were used to analyze the data of pH, VFA, and apparent nutrient digestibility. Alpha diversity indices were calculated using the “vegan“ package in R software (v4.3.2). Differential analysis of rumen microbiota composition, microbiota functions, and alpha diversity indices was conducted using the Wilcoxon rank-sum test on the Biozeron Cloud Platform (http://www.cloud.biomicroclass.com/CloudPlatform) [85]. The correlation analysis between rumen microbiota, pH, and VFA was calculated using the Spearman’s correlation test implemented in the “Hmisc” package in the R software (v4.3.2). Only significant correlations (*p* < 0.05) with a Spearman’s correlation coefficient|R| >0.6 were then visualized using Gephi software (v4.3.2) (https://gephi.org).

## Electronic supplementary material

Below is the link to the electronic supplementary material.

Supplementary Material 1

## Data Availability

All data generated and/or analysed during this study are available upon reasonable request to the corresponding author.

## Abbreviations

VFA: Volatile fatty acids
TVFA: Total volatile fatty acids
TMR: Total mixed ration
peNDF: Physically effective neutral detergent fiber
SARA: Subacute ruminal acidosis
NDF: Neutral detergent fiber
ADF: Acid detergent fiber
PSPS: Penn state particle separator
DMI: Dry matter intake
DM: Dry matter
OM: Organic matter
CP: Crude protein
EE: Ether extract
NFC: Non-fiber carbohydrate
ORFs: Open reading frames
KEGG: Kyoto encyclopedia of genes and genomes
CAZymes: Carbohydrate-active enzymes
KO: KEGG orthology
CPM: Counts per million
PCoA: Principal coordinates analysis
OTU: Operational taxonomic unit
AAs: Auxiliary activities
CBMs: Carbohydrate-binding modules
CEs: Carbohydrate esterases
GHs: Glycoside hydrolases
GTs: Glycosyltransferase
PLs: Polysaccharide lyases
PCA: Principal coordinate analysis
PEP: Phosphoenolpyruvate
Pta-AckA: Phosphate acetyltransferase-acetate kinase
