# Correction: Metagenomic analysis reveals rumen microbiome enrichment and functional genes adjustment in carbohydrate metabolism induced by different sorting behavior in mid-lactation dairy cows

**DOI:** 10.1186/s42523-025-00456-2

**Published:** 2025-08-25

**Authors:** Abdallah A. Mousa, Han Zhang, Hongwei Duan, Jiyou Zhang, Shengyong Mao

**Affiliations:** 1 Ruminant Nutrition and Feed Engineering Technology Research Center, College of Animal Science and Technology, Nanjing Agricultural University, Nanjing, 210095 Jiangsu China; 2 Laboratory of Gastrointestinal Microbiology, College of Animal Science and Technology, Nanjing Agricultural University, Nanjing, 210095 China; 3 Animal Production Department, Faculty of Agriculture, Benha University, Benha, 13736 Egypt

**Correction: anim microbiome 7**,** 82 (2025).**

10.1186/s42523-025-00439-3

Following publication of the original article [1], the authors reported that the name and family name of Abdallah A. Mousa had been mistakenly reversed. The corrected name provided in this Correction is accurate.

The original article [1] has been updated.
