# Supplementary material for: Metagenomic analysis reveals rumen microbiome enrichment and functional genes adjustment in carbohydrate metabolism induced by different sorting behavior in mid-lactation dairy cows
Source: Anim Microbiome. 2025 Jul 28;7:82. doi: 10.1186/s42523-025-00439-3 (PMC12302734; doi:10.1186/s42523-025-00439-3)
Supplement: Supplementary file 1 — Supplementary Material 1 [file 42523_2025_439_MOESM1_ESM.docx]

## ***Supplementary tables***

**Table S1** The difference in relative abundance of rumen bacteria at operational taxonomic units (OTU) levels (> 0.1% at least in one group; *P* < 0.05) between the SLS and SLS groups.

| Phylum | Genus | OTU | Relative abundance | | SEM | *P*-value |
| --- | --- | --- | --- | --- | --- | --- |
|  |  |  | SES | SLS |  |  |
| *Bacillota_A* | *Butyrivibrio* | *Butyrivibrio fibrisolvens* | 0.101 | 0.175 | 0.016 | 0.002 |
|  |  | *Butyrivibrio proteoclasticus* | 0.094 | 0.190 | 0.030 | 0.002 |
|  | *Papillibacter* | *Papillibacter cinnamivorans* | 0.116 | 0.216 | 0.017 | 0.002 |
|  | *Lachnobacterium* | *Lachnobacterium bovis* | 0.196 | 0.140 | 0.015 | 0.015 |
|  | *Pseudobutyrivibrio* | *Pseudobutyrivibrio xylanivorans* | 0.096 | 0.171 | 0.011 | 0.002 |
|  | *Eubacterium* | *Eubacterium Cellulosolvens* | 0.108 | 0.202 | 0.028 | 0.015 |
|  | *Saccharofermentans* | *Saccharofermentans acetigenes* | 0.207 | 0.133 | 0.018 | 0.015 |
|  |  | *Saccharofermentans sp902782125* | 0.252 | 0.128 | 0.023 | 0.002 |
|  | *Acetitomaculum* | *Acetitomaculum ruminis* | 0.177 | 0.114 | 0.016 | 0.004 |
|  | *Ruminococcus* | *Ruminococcus albus* | 0.130 | 0.222 | 0.029 | 0.015 |
|  |  | *Ruminococcus flavefaciens* | 0.123 | 0.225 | 0.025 | 0.015 |
| *Bacteroidota* | *Prevotella* | *Prevotella albensis* | 0.239 | 0.138 | 0.018 | 0.002 |
|  |  | *Prevotella sp002342415* | 0.175 | 0.098 | 0.018 | 0.002 |
|  |  | *Prevotella sp900314985* | 0.111 | 0.064 | 0.012 | 0.004 |
|  |  | *Prevotella sp017389705* | 0.221 | 0.120 | 0.028 | 0.008 |
|  |  | *Prevotella bryantii* | 0.241 | 0.145 | 0.027 | 0.015 |
|  |  | *Prevotella ruminicola* | 0.248 | 0.149 | 0.033 | 0.015 |
|  |  | *Prevotella brevis* | 0.199 | 0.116 | 0.027 | 0.025 |
|  |  | *Prevotella sp015060535* | 0.160 | 0.096 | 0.022 | 0.041 |
|  |  | *Prevotella sp902789415* | 0.198 | 0.126 | 0.067 | 0.041 |
|  | *RF16* | *RF16 sp900319035* | 0.178 | 0102 | 0.017 | 0.002 |
|  |  | *RF16 sp900320865* | 0.208 | 0.104 | 0.026 | 0.004 |
|  | *Sodaliphilus* | *Sodaliphilus sp902774055* | 0.180 | 0.134 | 0.063 | 0.041 |
| *Actinomycetota* | *Bifidobacterium* | *Bifidobacterium adolescentis* | 0.182 | 0.106 | 0.020 | 0.015 |
|  |  | *Bifidobacterium merycicum* | 0.164 | 0.095 | 0.021 | 0.015 |
| *Fibrobacterota* | *Fibrobacter* | *Fibrobacter succinogenes* | 0.111 | 0.191 | 0.020 | 0.002 |
| *Spirochaetota* | *Treponema* | *Treponema bryantii* | 0.246 | 0.153 | 0.029 | 0.025 |
|  |  | *Treponema saccharophilum* | 0.191 | 0.103 | 0.021 | 0.002 |
| *Bacillota* | *Lactobacillus* | *Lactobacillus acidophilus* | 0.194 | 0.119 | 0.017 | 0.004 |
| *Bacillota_C* | *Selenomonas* | *Selenomonas ruminantium* | 0.264 | 0.143 | 0.043 | 0.015 |
|  | *Anaeroplasma* | *Anaeroplasma bactoclasticum* | 0.115 | 0.198 | 0.027 | 0.025 |
|  |  | *Anaerosporobacter sp019421365* | 0.177 | 0.119 | 0.016 | 0.025 |
|  |  | *Anaeroplasma abactoclasticum* | 0.117 | 0.200 | 0.028 | 0.041 |
| *Pseudomonadota* | *Ruminobacter* | *Ruminobacter amylophilus* | 0.108 | 0.191 | 0.023 | 0.004 |
|  | *Acinetobacter* | *Acinetobacter baumannii* | 0.113 | 0.193 | 0.020 | 0.008 |

Differential analysis of rumen bacteria at the OUTs level was conducted using the Wilcoxon rank-sum test. n = 6.

## ***Supplementary figures***


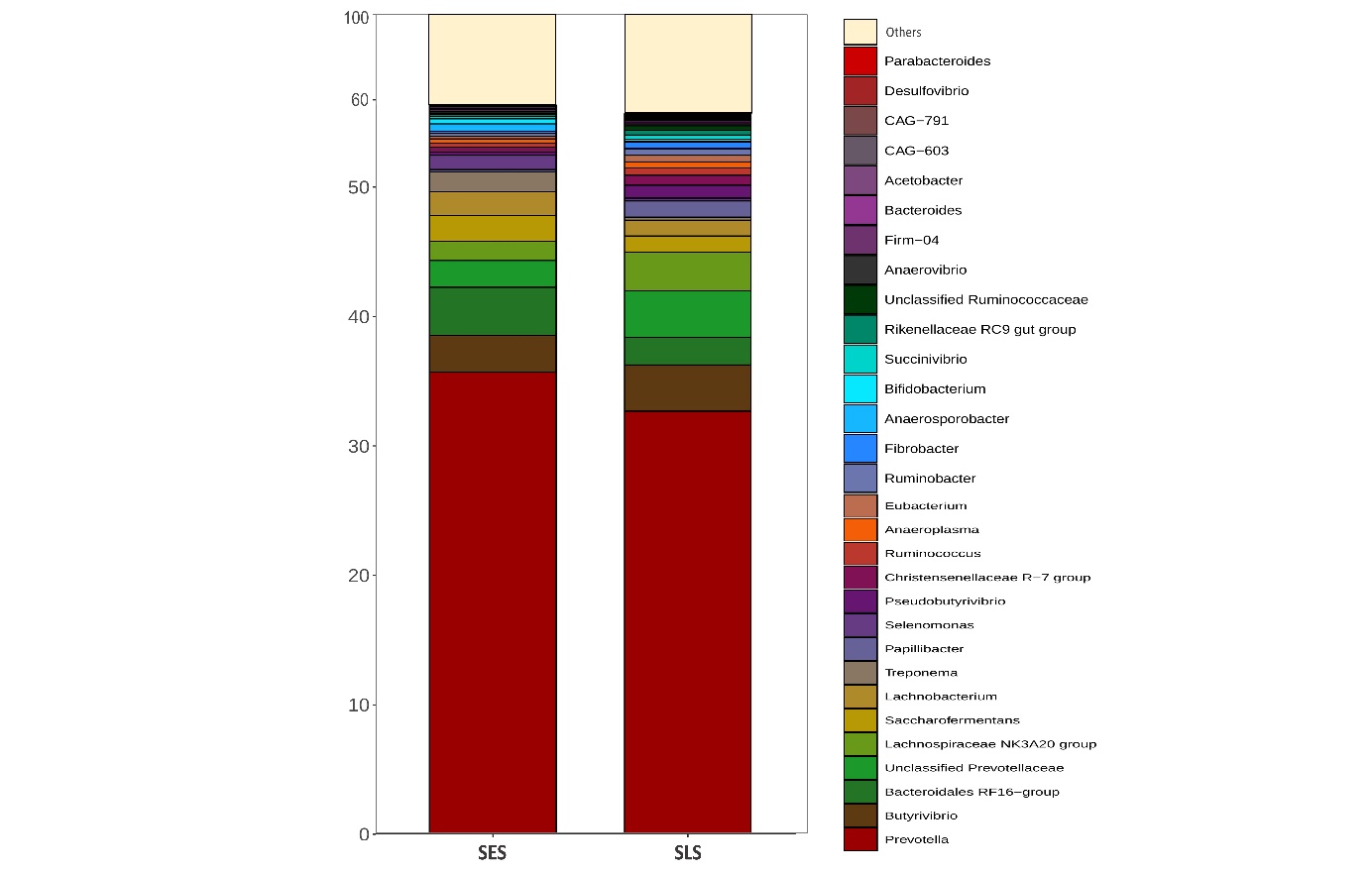


**Fig. S1** The relative abundance of rumen bacteria at genus level (> 0.1% in at least one group) between the SES and SLS groups. n = 6.


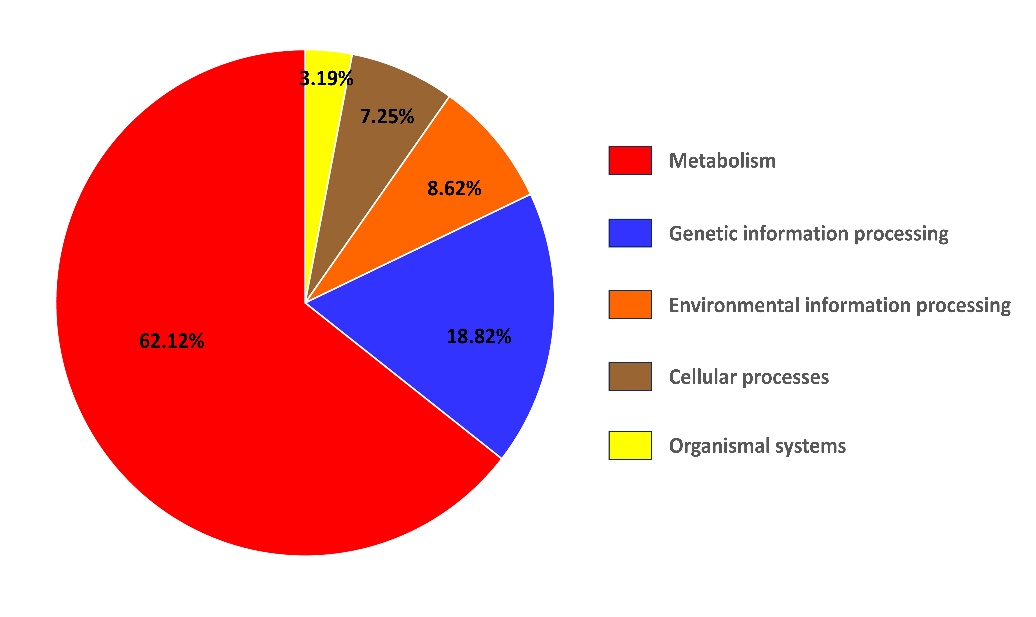


**Fig. S2** Overview of the average relative abundance of microbial KEGG modules at the first level of classification in the SES and SLS groups. n = 6.
